# Integration of Metabolome and Transcriptome Reveals the Relationship of Benzenoid–Phenylpropanoid Pigment and Aroma in Purple Tea Flowers

**DOI:** 10.3389/fpls.2021.762330

**Published:** 2021-11-23

**Authors:** Xin Mei, Shihua Wan, Chuyuan Lin, Caibi Zhou, Liuhong Hu, Chan Deng, Lingyun Zhang

**Affiliations:** ^1^College of Horticulture, South China Agricultural University, Guangzhou, China; ^2^College of Biological Science and Agriculture, Qiannan Normal University for Nationalities, Duyun, China

**Keywords:** *Camellia sinensis*, petal, anthocyanin, volatile, transcription factor, acetophenone, delphinidin-3-*O*-glucoside, transportation

## Abstract

Tea (*Camellia sinensis*) flowers are normally white, even though the leaves could be purple. We previously discovered a specific variety with purple leaves and flowers. In the face of such a phenomenon, researchers usually focus on the mechanism of color formation but ignore the change of aroma. The purple tea flowers contain more anthocyanins, which belong to flavonoids. Meanwhile, phenylalanine (Phe), derived from the shikimate pathway, is a precursor for both flavonoids and volatile benzenoid–phenylpropanoids (BPs). Thus, it is not clear whether the BP aroma was attenuated for the appearance of purple color. In this study, we integrated metabolome and transcriptome of petals of two tea varieties, namely, Zijuan (ZJ) with white flowers and Baitang (BT) with purple flowers, to reveal the relationship between color (anthocyanins) and aroma (volatile BPs). The results indicated that in purple petals, the upstream shikimate pathway promoted for 3-deoxy-D-arabino-heptulosonate 7-phosphate synthase (DAHPS) was elevated. Among the increased anthocyanins, delphinidin-3-*O*-glucoside (DpG) was extremely higher; volatile BPs, including benzyl aldehyde, benzyl alcohol, acetophenone (AP), 1-phenylethanol, and 2-phenylethanol, were also enhanced, and AP was largely elevated. The structural genes related to the biosynthesis of volatile BPs were induced, while the whole flavonoid biosynthesis pathway was downregulated, except for the genes *flavonoid 3′-hydroxylase* (*F3′H*) and *flavonoid 3′,5′-hydroxylase* (*F3′5′H*), which were highly expressed to shift the carbon flux to delphinidin, which was then conjugated to glucoside by increased bronze-1 (BZ1) (UDP-glucose: flavonoid 3-*O*-glucosyltransferase) to form DpG. Transcription factors (TFs) highly related to AP and DpG were selected to investigate their correlation with the differentially expressed structural genes. TFs, such as MYB, AP2/ERF, bZIP, TCP, and GATA, were dramatically expressed and focused on the regulation of genes in the upstream synthesis of Phe (*DAHPS*; *arogenate dehydratase/prephenatedehydratase*) and the synthesis of AP (*phenylacetaldehyde reductase*; *short-chain dehydrogenase/reductase*), Dp (*F3′H*; *F3′5′H*), and DpG (*BZ1*), but inhibited the formation of flavones (*flavonol synthase*) and catechins (*leucoanthocyanidin reductase*). These results discovered an unexpected promotion of volatile BPs in purple tea flowers and extended our understanding of the relationship between the BP-type color and aroma in the tea plant.

## Introduction

Benzenoids, especially phenylpropanoids, are the main sources of plant color and aroma. They help the plant to attract pollinators or deter enemies. Humans also gain health benefits such as antioxidation or improving memory (Cheng et al., 2016).

**FIGURE 1:**
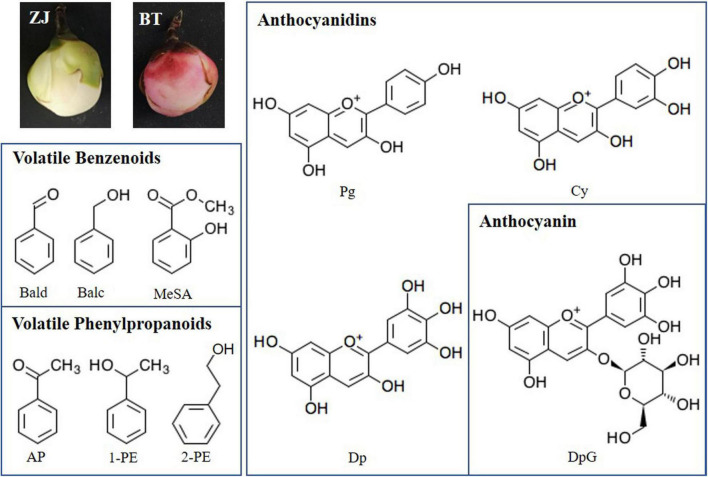
White [Zijuan (ZJ) variety] and purple [Baitang (BT) variety] tea flowers and structure of main benzenoid–phenylpropanoids (BPs) detected in this study. Bald, benzaldehyde; Balc, benzyl alcohol; MeSA, methyl salicylate; AP, acetophenone; 1-PE, 1-phenylethanol; 2-PE, 2-phenylethanol; Pg, pelargonidin; Cy, cyanidin; Dp, delphinidin; DpG, Dp-3-*O*-glucoside.

Benzenoids and phenylpropanoids (BPs) are a group of secondary metabolites originating from two aromatic amino acids phenylalanine (Phe) and tyrosine in the shikimic acid pathway. Phenylpropanoids contain an aromatic ring and a three-carbon propene tail. The simplest phenylpropanoid, cinnamic acid, is derived from the elimination of ammonia from Phe and transferred by cinnamate-4 hydroxylase (C4H) and 4-coumarate-CoA ligase (4CL); it leads to the biosynthesis of other phenylpropanoids, including flavonoids (Stevenson and Aslam, 2006). In flavonoid biosynthesis, chalcone is first generated by chalcone synthase (CHS) and then is isomerized to naringenin by chalcone isomerase (CHI). Naringenin is oxidized to dihydroflavonol by flavanone 3-hydroxylase (F3H). Flavonoid 3′-hydroxylase (F3′H) and flavonoid 3′,5′-hydroxylase (F3′5′H) add more hydroxyls to the secondary benzene ring of dihydroflavonol, which later direct to different types of catechins or anthocyanidins. Dihydroflavonol is reduced to leucoanthocyanidin, which is transferred to (+)-catechins and anthocyanidins by leucoanthocyanidin reductase (LAR) and anthocyanidin synthase (ANS), respectively. Anthocyanidins could be transferred to (−)-catechins by anthocyanidin reductase (ANR) or linked with glucose by UDP-glucose: flavonoid 3-*O*-glucosyltransferase (UFGT) to form anthocyanins. It has been widely proved that these structure genes were influenced by many transcription factors (TFs), such as *MYB*, *ERF*, *bZIP*, *TCP*, *GATA*, and so on (Wei et al., 2016; Chen et al., 2017; Jian et al., 2019; Gao et al., 2020; Fu et al., 2021). The important plant pigment anthocyanidins can be classified mainly into two groups, namely, flavonoids and phenolics. Anthocyanidins are generally conjugated with sugar molecules to form anthocyanins and accumulated in the vacuolar sap of the epidermal tissues. Anthocyanins can be divided into three subclasses, namely, anthocyanidin derivatives, non-acylated anthocyanidin glucoside, and acylated anthocyanidin glucoside (e.g., caffeoylated anthocyanin and malonylated anthocyanin) (Chandra Singh et al., 2020). The common anthocyanidin derivatives in plant include pelargonidin (Pg), cyanidin (Cy), delphinidin (Dp), peonidin (Pn), petunidin (Pt), and malvidin (Mv) (Chandra Singh et al., 2020). Moreover starting from cinnamate, common phenylpropanoid volatiles are synthesized, including phenylacetaldehyde, acetophenone (AP), 1-phenylethanol (1-PE), 2-phenylethanol (2-PE), and 2-phenylacetate. Other benzenoid volatiles are also generated from Phe but not *via* cinnamate, including benzaldehyde (Bald), benzyl alcohol (Balc), benzyl acetate, and methyl salicylate (MeSA) (Figure 1; Oliva et al., 2017). The diversity and richness of aroma are two reasons why tea [*Camellia sinensis* (L.) Kuntze] leaf has been made to be a popular non-alcoholic beverage. The main volatile BPs in tea leaves are 2-PE, Bald, Balc, and MeSA, while AP and 1-PE are unique in tea flower (Zhou et al., 2015; He et al., 2016). Volatile BPs and non-volatile BPs have a common upstream pathway. They both come from Phe, which is generated by the shikimic acid pathway. Phe enters Phe metabolism and Phe synthesis, respectively. The former pathway continues to form volatile BPs, and the latter forms flavonoids, including anthocyanins. The ideal material for studying BP pigments and aroma should preferably contain as many anthocyanins and volatile BPs as possible. Zijuan (ZJ; *C. sinensis* var. *kitamura*) is a special tea cultivar with purple buds, stems, and leaves, but it still lacked AP and 1-PE (He et al., 2016). Moreover, like other tea varieties, the flowers of ZJ are also white, which means low content of anthocyanins. Fortunately, we found a perfect material in the tea plantation of Baitang town, Boluo county, Guangdong province, China. Just near the ZJ, a natural mutant Baitang (BT; *C. sinensis* var. Baitang) has purple leaves and flowers (Zhou et al., 2020). These beautiful pink tea flowers provide a better model for studying the metabolism of BPs in tea plant and the relationship between color and scent caused by BPs. In our previous article, the developmental process of tea flower was divided into five stages, including mature flower bud (Stage 1), preopening (Stage 2), initial opening (Stage 3), half bloom (Stage 4), and full bloom (Stage 5). We found that total anthocyanin contents were similar between Stage 2 and Stage 3 and between Stage 4 and Stage 5, and anthocyanin had been generated in S1. To distinguish the obvious differences between stages, this time, we combined the similar stages defined last time and redivided tea flowers into three stages, namely, mature flower bud (Stage 1), before blooming (Stage 2), and blooming (Stage 3). In addition, the floral aroma will generally be released in large quantities after blooming. Considering the formation of anthocyanin in early stages and the amount of aroma, Stage 2 was thus selected as the experimental object in this study.

In this study, to discover the contribution of volatile and non-volatile BPs to the scent and color in purple tea flowers, BP-type aroma and anthocyanins were determined in white (ZJ variety) and purple (BT variety) tea petals (Figure 1), gene expression profile in the relevant pathways of biosynthesis and anthocyanin transportation were identified, and possible TFs for regulating these changes between ZJ and BT were analyzed.

## Materials and Methods

### Sample Preparation

The pink-flowered BT (*C. sinensis* L. var. Baitang) and the white-flowered ZJ (*C. sinensis* L. var. *kitamura*) were cultured in the tea plantation of Baitang town, Guangdong, China. Their fresh flowers of Stage 2 (preopening) were plucked, and the petals were collected and immediately fixed in liquid nitrogen on December 12, 2019. Each variety had three biological replicates. Each replicate was then ground in liquid nitrogen and stored at −80°C for standby.

### Transcriptome

Transcriptome of tea petals was sequenced and analyzed as described earlier (Zhou et al., 2020). In brief, 1 g of petals was extracted by using Sangon Total RNA Purification Kit (Shanghai Sangon Biotechnology Co., Ltd., Shanghai, China). The total RNA was sequenced and assembled on the Illumina HiSeq 2500 platform by the Biomarker Technologies Corporation (Beijing, China). The raw data have been uploaded to National Genomics Data Center, China, National Center for Bioinformation (NGDC, CNCB) with an accession number CRA005021.^1^ The clean reads were mapped and annotated following the reference genome (Wei et al., 2018) on TPIA website^2^ by using HISAT2 program (Xia et al., 2019). The levels of gene expression were estimated by Transcripts Per Kilobase of exon model per Million mapped reads (TPM) method. The significant difference between the two varieties was examined by DESeq2 R package (1.10.1). Differentially expressed genes were filtered by selecting a fold change greater than 2 and a false discovery rate less than 0.01.

### Quantitative Real-Time PCR

The quantitative real-time PCR (qRT-PCR) was performed as described before (Zhou et al., 2020). Briefly, total RNA was extracted from petals by using RNAqueous™ Total RNA Isolation Kit (Thermo, MA, United States). Primers of selected gene members were designed with the Primer Premier 5.0 software and listed in Supplementary Table 4. β-Actin was used as the reference gene. The fluorescence PCR reagent was the Hieff™ qPCR SYBR Green Master Mix (No Rox) (Yaesen Biotech Co., Ltd., Shanghai, China). The experiment and analysis were carried out on the LightCycler^®^ 480 II Real-Time System (Roche, CA, United States). Metabolome was carried out by using liquid chromatography–mass spectrometry.

The determination of the non-volatile metabolome of tea petals was described as earlier (Zhou et al., 2020). Briefly, 100 mg powder samples were extracted in 1.0 ml methanol (70%) at 4°C for 24 h, and 5 μl supernatant was injected into ultra-performance liquid chromatography (UPLC, Shimadzu Co., Kyoto, Japan) with a mass system (MS, Applied Biosystems 6500 Q TRAP, MA, United States). Metabolites were identified using MWDB (Metware Database, Metware Biotechnology Co., Ltd., Wuhan, China) and subject to the partial least squares (PLS) discriminant analysis. The significant dissimilarities of metabolites were set as the variable importance (VIP) ≥1 and the fold change ≥2 or ≤0.5.

### Volatile Profile

Two grams of frozen samples were accurately weighed and heated in an air bath at 45°C for 5 min. Then, a manual injector with 65 μm polydimethylsiloxane/divinylbenzene extraction head was inserted to conduct headspace extraction under the condition of 45°C air bath. After 40 min of extraction, the manual injector was inserted into the injection port of the gas chromatograph–mass spectroscopy (GC–MS) immediately, and the instrument started to collect data at the same time. GC column: DB-5MS (30 m × 250 μm × 0.25 μm); injection port temperature: 250°C; carrier gas: helium (99.999%); flow rate: 1.6 ml/min; temperature program: 50°C for 3 min, then increasing to 265°C at the speed of 4.0°C/min, and keeping for another 5 min. MS ionization mode: EI; electron energy: −70 eV; quality scanning range: 33–600 U; ion source temperature: 220°C. The mass spectrum data obtained from GC–MS analysis were searched in NIST98.L standard spectrum library. The relevant mass spectrum data were checked, and the base peak, mass nucleus ratio, and relative abundance were analyzed. The structures and names of aromatic compounds represented by each peak were confirmed. The relative content of components is obtained by the ratio of the peak area of each aroma component to the total peak area.

### Statistical Analysis

Pearson’s correlation between the content of metabolites and the levels of gene expression was analyzed by using R (version 4.0.3). The correlation network was drawn by Cytoscape (v3.8.2). Other figures were presented by Excel 2010.

## Results

### Volatile Benzenoid–Phenylpropanoids and Related Genes

**FIGURE 2:**
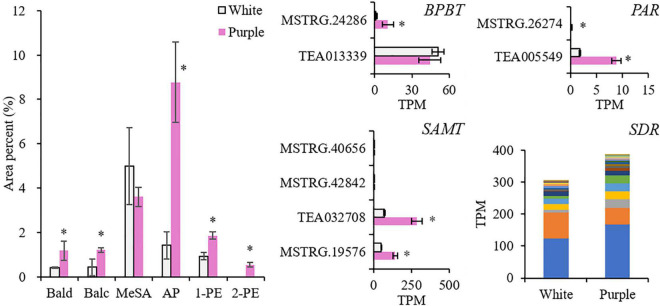
Contents of volatile benzenoid–phenylpropanoids (BPs) and related genes in white [Zijuan (ZJ) variety] and purple [Baitang (BT) variety] tea flowers. The stacked columns of *SDR* contain the gene members with significant difference between the two tea varieties. *Indicates significant difference (*p* < 0.05) between the white and the purple. Bald, benzaldehyde; Balc, benzyl alcohol; MeSA, methyl salicylate; AP, acetophenone; 1-PE, 1-phenylethanol; 2-PE, 2-phenylethanol; BPBT, benzoyl-CoA:benzyl alcohol/2-phenylethanol benzoyltransferase; SAMT, salicylic acid carboxyl methyltransferase; PAR, phenylacetaldehyde reductase; SDR, short-chain dehydrogenase/reductase.

The most abundant volatile BPs in both the white and purple tea flowers were MeSA and two phenylpropanoid volatiles, namely, AP and 1-PE (Figure 2, Supplementary Figure 1, and Supplementary Table 1). MeSA was the dominant volatile BPs in the white flowers of ZJ, while in the purple flowers of BT, it was AP, which was 6.2 times higher than that in ZJ. Except for MeSA, other common volatile BPs were significantly elevated in purple flowers: 1-PE, Bald, and Balc were 2, 2.9, and 2.8 times higher, respectively; and 2-PE was even not detected in white flowers. In terms of the total amounts, the dominant aroma from BPs was changed in the purple tea flowers, where the phenylpropanoids (AP, 1-PE, and 2-PE) were higher than other benzenoids (Bald, Balc, and MeSA), while in the normal whiter flowers, it was opposite. It indicated that the synthetic pathways of phenylpropanoids were promoted in the purple tea flowers.

Then, we investigated four enzyme genes responsible for the direct synthesis of benzyl benzoate, MeSA, 2-PE, and AP, which were benzyl alcohol *O*-benzoyltransferase (BPBT; KO id is K19861), salicylic acid carboxyl methyltransferase (SAMT; KO id is K21483), phenylacetaldehyde reductase (PAR), and short-chain dehydrogenase/reductase (SDR), respectively (Zhou et al., 2015; Cheng et al., 2016). They were all enhanced in the purple petals. A stacked column was used to illustrate the 22 gene members of *SDR* with significant differences between the two varieties. The sum of the 22 *SDR* members was 1.3-fold higher in the purple petals and so was the member (TEA025281) with the highest expression level. *BPBT* and *PAR* had low expression levels but were 15 and 5 times higher, respectively. Two members of *SAMT* were highly induced in tea flowers, and they were 3–4 times higher in purple petals. These results proved that the biosynthesis of volatile BPs was stronger in the purple tea flowers.

### Non-volatile Benzenoid–Phenylpropanoids and Related Genes

**FIGURE 3:**
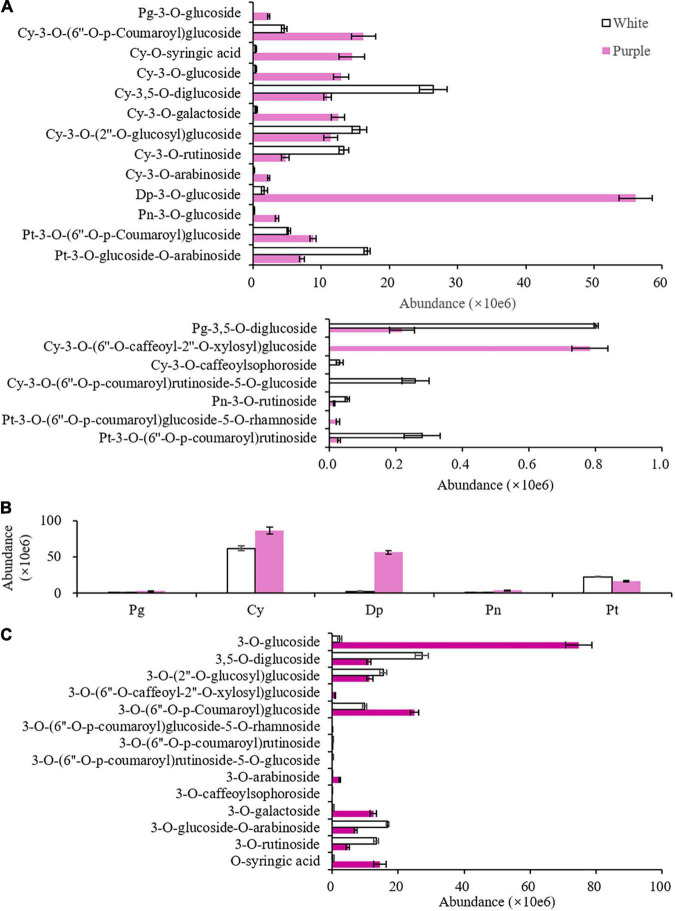
Non-volatile benzenoid–phenylpropanoids (BPs) with significant difference (*p* < 0.05) between the white [Zijuan (ZJ) variety] and purple [Baitang (BT) variety] petals of tea plants. **(A)** Peak area of all anthocyanins. **(B)** Aggregation of the same aglycone. **(C)** Aggregation of the same glycosyl. Pg, pelargonidin; Cy, cyanidin; Dp, delphinidin; Pn, peonidin; Pt, petunidin.

A total of 20 anthocyanins were detected in the petals of the 2 varieties, and 5 anthocyanidins were found as aglycones, namely, Pg, Cy, Dp, Pn, and Pt. Mv did not exist in tea. Each variety had two or three undetected anthocyanins, namely, petunidin-3-*O*-(6″-*O*-*p*-coumaroyl)glucoside-5-*O*-rhamnoside, cyanidin-3-*O*-(6″-*O*-caffeoyl-2″-*O*-xylosyl)glucoside, and pelargonidin-3-*O*-glucoside in ZJ and cyanidin-3-*O*-(6″-*O*-*p*-coumaroyl)rutinoside-5-*O*-glucoside and cyanidin-3-*O*-caffeoylsophoroside in BT (Figure 3A and Supplementary Table 2). The total content of these 20 anthocyanins was 1.9 times higher in the purple petals than that in the white petals. According to the types of aglycones, 20 anthocyanins can be divided into 2 Pg, 11 Cy, 1 Dp, 2 Pn, and 4 Pt, of which Cy and Dp were the dominant aglycones (Figure 3B). Although Dp has only one glycosidic form, it was increased largely in the purple petals. The dominant anthocyanins in the purple petals were delphinidin-3-*O*-glucoside (DpG, Mirtillin), Cy-3-*O*-(6″-*O*-*p*-coumaroyl)glucoside, Cy-*O*-syringic acid, Cy-3-*O*-glucoside (Kuromanin), and Cy-3-*O*-galactoside, while in white flowers, they were Cy-3,5-*O*-diglucoside (Cyanin), Pt-3-*O*-glucoside-*O*-arabinoside, Cy-3-*O*-(2″-*O*-glucosyl)glucoside, and Cy-3-*O*-rutinoside (Keracyanin). Apart from aglycones, the other half of these 20 anthocyanins contained 13 glycosides and 1 organic acid, which was syringic acid, a member of benzoic acids (Figure 3C). In white petals of ZJ, the most anthocyanins were 3,5-*O*-diglucosides, and in purple petals of BT, 3-*O*-glucosides were extremely elevated. As a result, the biggest difference of anthocyanins between the two tea varieties was DpG, which might be the main reason for the purple color of the BT flowers, and the biosynthesis of Dp and glucose had probably been enhanced in the purple petals.

**FIGURE 4:**
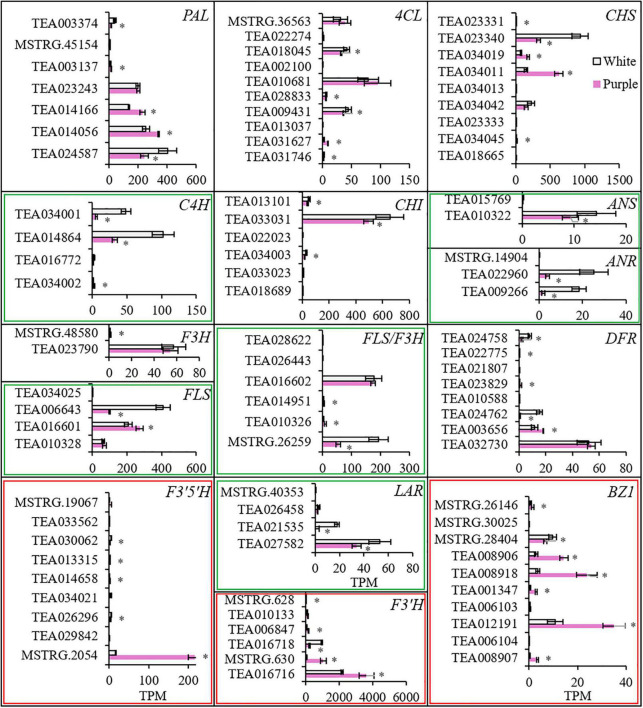
The expression levels of genes involved in biosynthesis of anthocyanins. *Indicates significant difference (*p* < 0.05) between the white and the purple. PAL, phenylalanine ammonia lyase; 4CL, 4-coumarate-CoA ligase; C4H, cinnamate-4 hydroxylase; CH, chalcone synthase; CHI, chalcone isomerase; F3H, flavanone 3-hydroxylase; F3′H, flavonoid 3′-hydroxylase; F3′5′H, flavonoid 3′,5′-hydroxylase; FLS, flavonol synthase; DFR, dihydroflavonol 4-reductase; LAR, leucoanthocyanidin reductase; ANS, anthocyanidin synthase; ANR, anthocyanidin reductase; BZ1, bronze-1 (i.e., anthocyanidin 3-*O*-glucosyltransferase or UFGT).

To investigate the enhanced biosynthesis progresses for producing anthocyanins, the transcriptomes of petals were sequenced for ZJ and BT. The expression profiles of genes involved in flavonoid biosynthesis pathway showed that each gene had several members, and some were higher in white petals and others were converse (Figure 4 and Supplementary Table 3). However, the sum of the expression of members with significant differences in each gene indicated that most genes in purple petals were downregulated, especially *C4H*, *FLS*, *LAR*, and *ANR*, which were largely decreased. But *F3′H* and *F3′5′H* were significantly upregulated. In particularly, *F3′5′H* was elevated by eight times, which proved its critical role in generating Dp. Moreover, due to the little amount of *ANR*, Dp could not be reduced to epigallocatechin and thereby fluxed into the synthesis of DpG, which was catalyzed by bronze-1 (BZ1). BZ1 is an anthocyanidin 3-*O*-glucosyltransferase, also known as UFGT. The total expression level of *BZ1* gene members was three times higher in the purple petals than that in the white.

**FIGURE 5:**
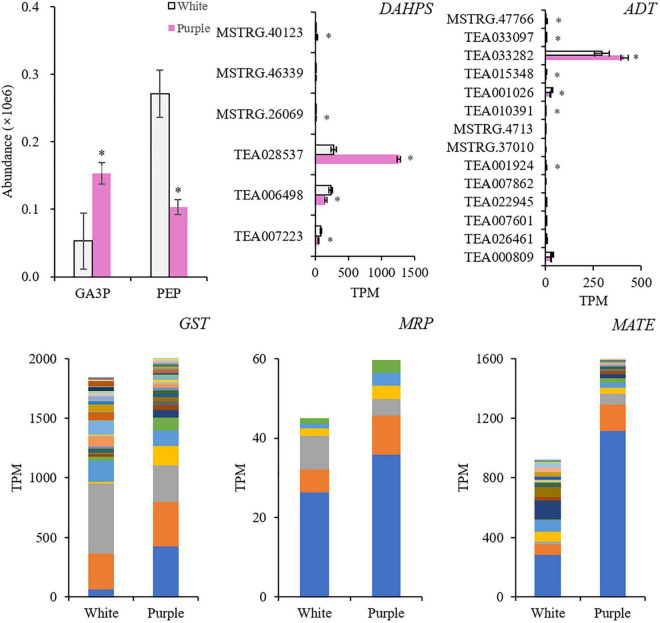
Key compounds and genes in the upstream biosynthetic pathways of benzenoid–phenylpropanoids (BPs), and the genes related to the transportation of anthocyanins. *Indicates significant difference (*p* < 0.05) between the white and the purple. GA3P, glyceraldehyde 3-phosphate; PEP, phosphoenolpyruvate; DAHPS, 3-deoxy-D-arabino-heptulosonate 7-phosphate synthase; ADT, arogenate dehydratase/prephenate dehydratase; GST, glutathione *S*-transferase; MRP, multidrug resistance-associated protein; MATE, multidrug and toxin extrusion.

To further reveal the different synthetic mechanisms of BPs between the two tea varieties, the upstream compounds and genes were detected (Figure 5). In purple petals, glyceraldehyde 3-phosphate (GA3P) increased 2.9 times, while phosphoenolpyruvate (PEP) was only 38% of that in the white petals. It indicated that the carbon metabolism in the purple petals was promoted, but it might not flux into the downstream metabolism of Phe. The decrease of PEP might be due to the highly induced enzyme 3-deoxy-D-arabino-heptulosonate 7-phosphate synthase (DAHPS). The main member of *DAHPS* gene TEA028537 was 4.6-fold higher in the purple petals. Arogenate dehydratase/prephenate dehydratase (ADT) catalyzes arogenate to produce Phe. Its main member TEA033282 was elevated a little in purple petals, which was 1.3-fold higher. In addition, synthesized anthocyanins need to be transported to vacuole before displaying color. This progress is related to glutathione *S*-transferase (GST), multidrug resistance-associated protein (MRP), and multidrug and toxin extrusion (MATE). The expression levels of these gene members with significant differences were added up, and the total amount was all higher in the purple petals, which were 1.1, 1.3, and 1.7 times higher, respectively. The main members (TEA015341, TEA013531, and TEA006958) of these three genes in the purple petals were induced 6.9, 1.4, and 3.9 times higher, respectively.

### Transcription Factors Involved in the Biosynthesis of Benzenoid–Phenylpropanoids

The results above suggested that in purple flowers, the most characteristic volatile and non-volatile BPs were AP and DpG, respectively. The Pearson’s correlation coefficients were calculated between these two BPs and the TFs, which were significantly different between the two tea varieties. There were 14 TFs whose correlation coefficients were greater than 0.95 both with AP and DpG (Table 1). They belong to the families of *AP2/ERF*, *bZIP*, *EMP*, *GATA*, *HS*, *MYB*, *NAC*, *TCP*, and *WRKY* and were 1.6–6.7 times higher in the purple petals. *MYB*, *AP2/ERF*, *bZIP*, *TCP*, and *GATA* had the most expression levels.

**TABLE 1:** The candidate transcription factors (TFs) involved in the biosynthesis of acetophenone (AP) and delphinidin-3-*O*-glucoside (DpG).

TF family	Gene ID	Expression level (TPM)		Correlation coefficient		Annotation
		ZJ	BT	AP	DpG	
AP2/ERF	TEA030964	9.9 ± 1.0	24.1 ± 3.2	0.962	0.965	APETALA2 (*Camellia sinensis*)
	TEA004341	4.4 ± 0.4	8.9 ± 1.3	0.979	0.960	ERF118-like (*Nicotiana sylvestris*)
bZIP	TEA032800	12.5 ± 1.2	19.8 ± 1.4	0.956	0.963	bZIP 18 (*Camellia sinensis*)
	TEA014041	0.7 ± 0.2	1.5 ± 0.2	0.968	0.952	TGA2.3 (*Vitis vinifera*)
EMB	MSTRG.32209	N.D.	1.6 ± 0.2	0.956	0.983	EMB1444-like (*Camellia sinensis*)
GATA	TEA027958	N.D.	0.4 ± 0.0	0.957	0.996	GATA (*Actinidia chinensis* var. *chinensis*)
	TEA022802	2.1 ± 0.8	13.5 ± 2.5	0.954	0.962	GATA 26-like (*Nicotiana tabacum*)
HS	TEA000588	1.1 ± 0.0	1.8 ± 0.2	0.971	0.953	Heat stress TF B-3-like (*Quercus suber*)
MYB	TEA024999	0.9 ± 0.2	2.5 ± 0.3	0.970	0.972	PHL5 (*Vitis vinifera*)
	MSTRG.43318	4.4 ± 0.6	15.3 ± 1.7	0.969	0.983	ETC3 (*Camellia sinensis*)
	TEA002399	7.7 ± 0.6	26.7 ± 1.6	0.951	0.997	MYB61 (*Arabidopsis thaliana*)
NAC	MSTRG.28161	N.D.	5.0 ± 1.2	0.987	0.973	NAC 29-like (*Camellia sinensis*)
TCP	TEA009154	3.2 ± 1.2	15.5 ± 2.6	0.962	0.976	TCP4 (*Glycine max*)
WRKY	TEA012360	0.2 ± 0.1	1.3 ± 0.3	0.984	0.959	WRKY 40 (*Actinidia chinensis* var. *chinensis*)

**FIGURE 6:**
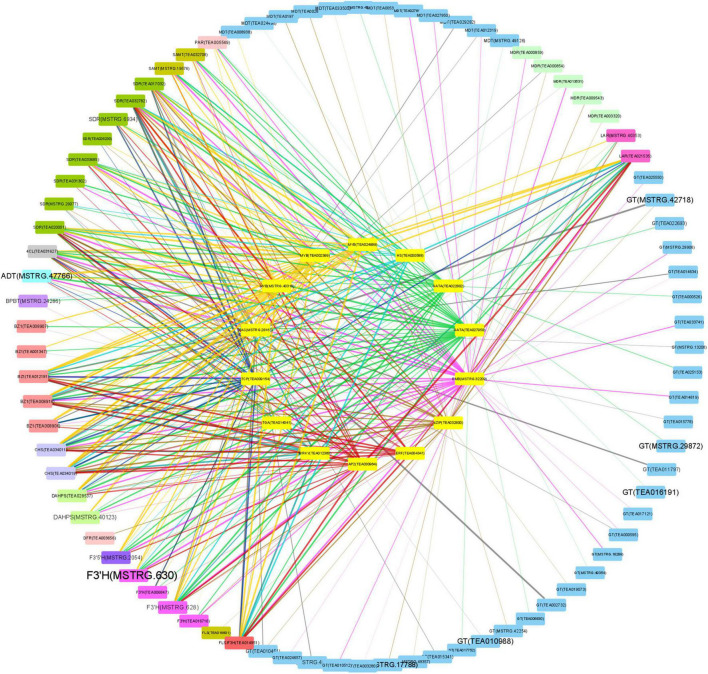
Correlations between transcription factors (TFs) and structural genes. Big circle: structural genes; small circle: TFs; color of nodes: gene types; font size of structural genes: fold change of Baitang (BT) compared to Zijuan (ZJ); color of edges: TF types; width of edges: correlation coefficient.

Correlations between the 14 TFs and the structural genes narrated above were analyzed to discover the potential regulatory mechanisms (Figure 6). Most of these TFs are highly related to *PAR*, *SAMT*, and *SDR*, which contributed to the biosynthesis of volatile BPs; and *4CL*, *BZ1*, *CHS*, *F3′H*, *F3′5′H*, *FLS*, and *LAR*, which were responsible for non-volatile BPs; and *ADT* and *DAHPS* in the upstream biosynthesis of Phe. By contrast, these TFs did not focus on a specific gene for anthocyanin transportation, neither on BPBT in the biosynthesis of benzyl benzoate and *PAL*, *C4H*, *CHI*, *ANS*, *ANR*, *F3H*, and *DFR* in the pathway of flavonoid biosynthesis.

### Validation of Gene Expression Levels by Real-Time PCR

**FIGURE 7:**
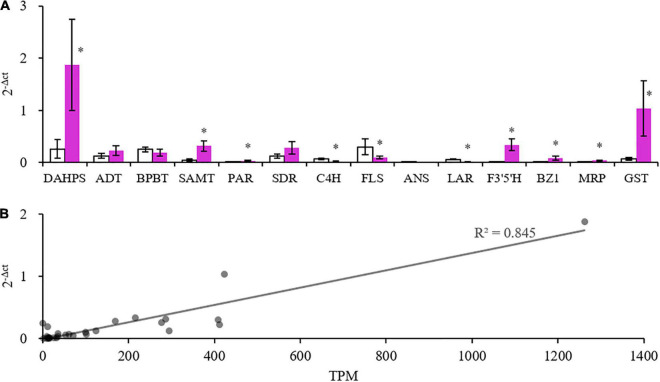
Quantitative real-time polymerase chain reaction (PCR) (qRT-PCR) validation of some selected genes. **(A)** Expression values of genes. *Indicates significant difference (*p* < 0.05) between the white and purple petals. **(B)** Correlation of qRT-PCR results and Transcripts Per Kilobase of exon model per Million mapped reads (TPM) values of the selected genes.

Some gene members with high expression levels determined by RNA-seq were selected (Supplementary Table 4) to be further validated by real-time PCR. The expression profiles of the two methods were consistent, and the correlation coefficient between 2^–Δ^ *^ct^* and TPM was 0.845, showing a good correlation (Figure 7). These results indicated that the transcriptome data were credible.

## Discussion

The floral color of common *Camellia*, such as *C. reticulata* and *C. sasanqua*, ranges from white to red. However, the flowers of tea have been observed to be white in the past (Chen et al., 2020), though it has purple leaves. Exceptionally, a new mutation with purple flowers was found recently, which provides an opportunity for us to study the color formation in tea plant. The purple color is from the pigment of anthocyanins (Liu et al., 2016). Furthermore, floral scents are different from the aroma emitting from tea leaves, such as AP, a major fragrance in tea flowers (Zhou et al., 2015). Because anthocyanins and AP are all generated from Phe metabolism, it attracted us to find out whether the occurrence of purple color leads to the attenuation of aroma in the related pathways.

**FIGURE 8:**
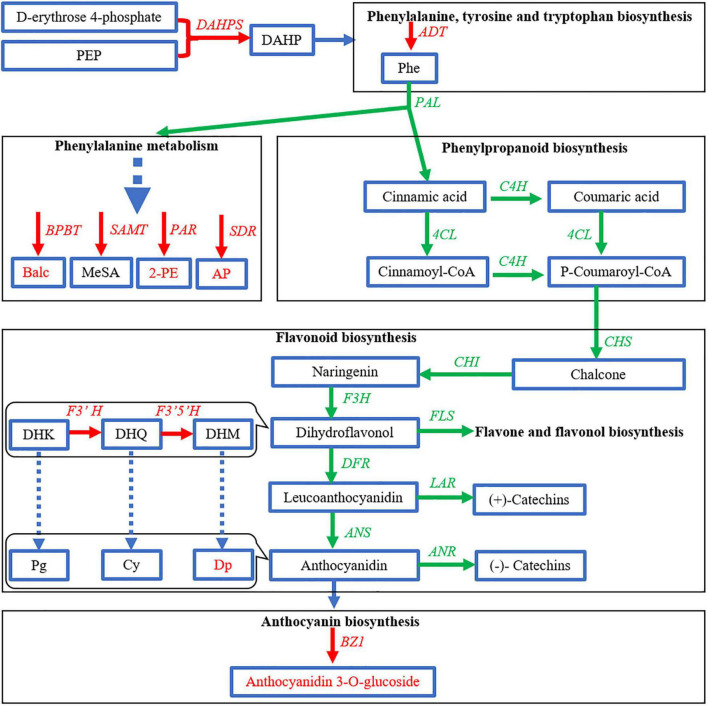
Expression profile of genes related to biosynthesis of benzenoid–phenylpropanoids (BPs) in purple petals [Baitang (BT) variety] compared to that in white petals [Zijuan (ZJ) variety]. Green and red arrows indicate lower and higher in the purple petals, respectively.

Carbohydrate metabolism was extremely important for the biosynthesis of flavonoids. The production of PAL and flavonoids in strawberry leaves depended on a supply of carbon dioxide (Creasy, 1968). Herein, the increase of GA3P in purple petals indicated the stimulation of carbon flux exchange. The induction of *DAHPS* and *ADT* further suggested that the shikimate pathway had been directed to produce Phe. The shikimate pathway links the primary metabolism and the downstream biosynthesis of BPs (Oliva et al., 2017). However, the decrease of PEP in the upstream pathway and the downregulation of genes in the main route of phenylpropanoid and flavonoid biosynthesis showed no obvious evidence that a large quantity of carbon flux had entered the pathway to produce BPs. The stimulated carbon metabolism might mainly contribute to gluconeogenesis and pentose phosphate pathway to synthesize the glycosyl moiety of anthocyanins. In flavonoid biosynthesis pathway, only *F3′H* and *F3′5′H* were elevated to increase the downstream Cy and Dp. In tea flowers, whether in white or purple color, Cy was conjugated to several carbohydrates, while Dp was only conjugated to glucoside. But the contents of total Cy and its glycosides in white petals were close to that in the purple petals. Thus, as *BZ1* was induced, Dp-3-*O*-glucoside became the mainly increased and dominant anthocyanin in purple petals. Although the carbon flux might not increase, it seems that anthocyanins and flavonoids are always in different channels. The carbon flux in dark-exposed *Begonia semperflorens* channeled into flavonoids from anthocyanins (Zhang et al., 2016). We also found the inhibition of *FLS*, *LAR*, and *ANR* in the branching way to produce flavones and flavanols (catechins). Taken together, anthocyanins in purple petals were probably due to the shift of carbon flux from flavones and flavanols but little from upstream pathways (Figure 8). The promoted upstream pathway, i.e., the shikimate pathway, might flux into other aromatic derivatives, such as volatile BPs. Although *F′3′5′H* and *BZ1* had critical effects on the formation of anthocyanins, the highly expressed gene members were different from the identified members in tea leaf (Zhao et al., 2017; Su et al., 2018; Guo et al., 2019).

As for the volatile BPs, the contents of aroma and related genes were increased in the purple petals, except for a few indexes (e.g., MeSA) with no significant difference. It means that the biosynthesis pathways of volatile BPs were generally promoted but not reduced with the increasing purple color as we thought before. It was similar in purple cherry tomato, where the types of volatile compounds were less than that in red and orange tomatoes, but phenolic volatiles increased (Silva Souza et al., 2020). Whether white tea flowers smell better needs a more comprehensive investigation. In tea leaves, single blue or red light-induced *PAL* gene and volatile benzenoids but not volatile phenylpropanoids (Fu et al., 2015). It seemed that *PAL* might only affect benzenoid aroma. However, the findings of this study showed that BPs increased in purple flowers despite the downregulation of *PAL*, which means *PAL* may not be a decisive factor. Volatile phenylpropanoids are special aroma in tea flower. In particular, 1-PE and AP are highly accumulated in tea flower but not in tea leaf and increase during floral development (Chen et al., 2018). In the white petals of ZJ variety, 1-PE and AP were indeed the main phenylpropanoid aroma. However, they were surprisingly even much higher in the purple petals of BT variety, and AP became the highest volatile BPs, which obviously improved the aroma characteristics of tea flower. So far, the formation mechanism of AP has been not very clear. The increased 1-PE was probably the precursor/metabolite of AP, and SDR might be the potential enzyme (Zhou et al., 2015), but its members did not show as much change as AP. The reason for the increase of AP in purple tea flowers is worth further investigation.

Furthermore, from the results, we could see that anthocyanins also existed in the white petals. Except for the concentration, the color was affected by pH and anthocyanin transportation into vacuole. Three types of mediation have been proposed for anthocyanin transport: GST, membrane transporter, and membrane vesicle (Zhao et al., 2020). GST sequestrates anthocyanin from cytosol to the vacuole. *PpGST1* correlated well with anthocyanin accumulation in peach fruit (Zhao et al., 2020). The second mechanism relies on MATE transporters and ABC transporters located in the tonoplast. *ZmMRP3* was first reported as an anthocyanin transporter and controlled by the regulators of anthocyanin biosynthesis (Goodman et al., 2004). The flower color of Asiatic Hybrid Lilies (*Lilium* spp.) is half purple (lower part) and half white (upper part), which is similar to BT flowers. An MATE gene *LhDTX35* might function as a carrier protein to transport anthocyanins (Xu et al., 2020). Furthermore, a flavonoid carrier similar to mammalian bilitranslocase (BTL) was identified in grape, and it may participate in long distance transport of flavonoids for its presence in vascular bundles (Petrussa et al., 2013). Thus, the transportation of anthocyanins is related to GST, MRP, MATE, and BTL-homolog. Herein, in tea flowers, GST, MATE, and MRP have been detected. Their main members and the total amount were higher in the purple petals, which indicated a promotion of anthocyanin transportation. Flavonoids in tea flowers may not be transported from tea leaves, since BTL-homolog was not detected in the flowers of both tea varieties. Many genes involved in the synthesis of flavonoids have been stimulated during the tea flower development (Chen et al., 2020). However, more experiments are needed to validate the sources of flavonoids in tea flower.

Since the biosynthetic pathways of volatile and non-volatile BPs are not fully understood, it is difficult to find a key enzyme or its gene as a hub gene controlling the synthetic direction of BPs for pigment or aroma. Nevertheless, we may get some clues from the analysis of TFs. In this study, we found 14 TFs were highly related to AP and DpG, of which *MYB*, *AP2/ERF*, *bZIP*, *TCP*, and *GATA* had relatively higher expression levels. They all play important roles in elevation of aroma and pigment. *SlMYB75* promoted accumulation of anthocyanin and volatile aroma production in tomato fruits (Jian et al., 2019). *AP2/ERF* regulates the expression of ethylene responsive genes in plant development and flower abscission (Gao et al., 2020). *bZIP* affected anthocyanin accumulation in apple and aroma in banana. *FvTCP9* dramatically promotes the genes involved in fruit color and aroma metabolism in strawberry fruits (Wei et al., 2016). *GATA* promoted crucial genes of Ehrlich pathway to enhance 2-PE production in *Saccharomyces cerevisiae* (Chen et al., 2017) and regulate anthocyanin accumulation in *Camellia japonica* petals (Fu et al., 2021). The 14 TFs herein were also highly related to the genes in the biosynthesis of Phe and BPs. This is in accordance with the fact that these TFs were highly related to the content of AP and DpG. These TFs focused on the genes for synthesis of AP (*PAR* and *SDR*), Dp (*F3′H* and *F3′5′H*), and DpG (*BZ1*) but inhibited the formation of flavones (*FLS*) and catechins (*LAR*). This indicated that in spite of the promotion of the upstream Phe synthesis (*ADT* and *DAHPS*), these TFs only changed the synthetic direction to Dp and blocked other flavonoids in the pathway of flavonoid biosynthesis.

## Conclusion

It is an interesting thing to know what happens to the aroma when the flower color mutates naturally. In the purple petals of BT tea flowers, the BP-type aroma increased as the BP-type color occurred. AP and DpG were largely accumulated and become the dominant volatile and non-volatile BPs. The structural genes in the shikimate pathway and the biosynthesis of volatile BPs were promoted. Most genes in flavonoid biosynthesis were downregulated, and the efflux was directed to accumulate Dp. Many TFs were involved in regulating the purple color and its related aroma. The outcome of this study revealed the relationship between the BP-type color and aroma in tea plant. To further clarify the regulatory mechanism, more identification of the functions of relevant genes will be needed in the future.

## Data Availability Statement

The datasets presented in this study can be found in online repositories. The names of the repository/repositories and accession number(s) can be found below: https://ngdc.cncb.ac.cn/gsa/browse/CRA005021, PRJCA006284.

## Author Contributions

LZ and XM designed the research. XM wrote the manuscript. SW, LZ, and XM collected the experimental materials. SW, CL, and LH conducted the experiment involved in this manuscript. CL, CZ, and CD helped with data analysis. LZ revised the manuscript critically for important intellectual content. All authors listed here contributed and approved the manuscript.

## Conflict of Interest

The authors declare that the research was conducted in the absence of any commercial or financial relationships that could be construed as a potential conflict of interest.

## Publisher’s Note

All claims expressed in this article are solely those of the authors and do not necessarily represent those of their affiliated organizations, or those of the publisher, the editors and the reviewers. Any product that may be evaluated in this article, or claim that may be made by its manufacturer, is not guaranteed or endorsed by the publisher.

## Footnotes

1
https://bigd.big.ac.cn/gsa/browse/CRA005021

2
http://tpdb.shengxin.ren/

## Supplementary Material

Supplementary Figure 1 Total ion chromatogram (TIC) of tea flower from gas chromatography-mass spectroscopy (GC–MS).

## Abbreviations

BPs: benzenoids and phenylpropanoids
1-PE: 1-phenylethanol
2-PE: 2-phenylethanol
4CL: 4-coumarate-CoA ligase
ADT: arogenate dehydratase/prephenate dehydratase
ANR: anthocyanidin reductase
ANS: anthocyanidin synthase
AP: acetophenone
Balc: benzyl alcohol
Bald: benzaldehyde
BPBT: benzyl alcohol *O*-benzoyltransferase
BT: Baitang
BZ1: bronze-1
C4H: cinnamate-4 hydroxylase
CHI: chalcone isomerase
CHS: chalcone synthase
Cy: cyanidin
DAHPS: 3-deoxy-D-arabino-heptulosonate 7-phosphate synthase
DFR: dihydroflavonol 4-reductase
Dp: delphinidin
DpG: delphinidin-3-*O*-glucoside
F3′5′H: flavonoid 3′ ,5′ -hydroxylase
F3H: flavanone 3-hydroxylase
F3′H: flavonoid 3′ -hydroxylase
FLS: flavonol synthase
GA3P: glyceraldehyde 3-phosphate
GST: glutathione *S*-transferase
LAR: leucoanthocyanidin reductase
MATE: multidrug and toxin extrusion
MeSA: methyl salicylate
MRP: multidrug resistance-associated protein
Mv: malvidin
PAR: phenylacetaldehyde reductase
PEP: phosphoenolpyruvate
Pg: pelargonidin
Phe: phenylalanine
Pn: peonidin
Pt: petunidin
SAMT: salicylic acid carboxyl methyltransferase
SDR: short-chain dehydrogenase/reductase
TFs: transcription factors
TPM: Transcripts Per Kilobase of exon model per Million mapped reads
UFGT: UDP-glucose: flavonoid 3-*O*-glucosyltransferase
ZJ: Zijuan.
