# Supplementary figures and images for: Integration of Metabolome and Transcriptome Reveals the Relationship of Benzenoid–Phenylpropanoid Pigment and Aroma in Purple Tea Flowers
Source: Front Plant Sci. 2021 Nov 23;12:762330. doi: 10.3389/fpls.2021.762330 (PMC8649654; doi:10.3389/fpls.2021.762330)

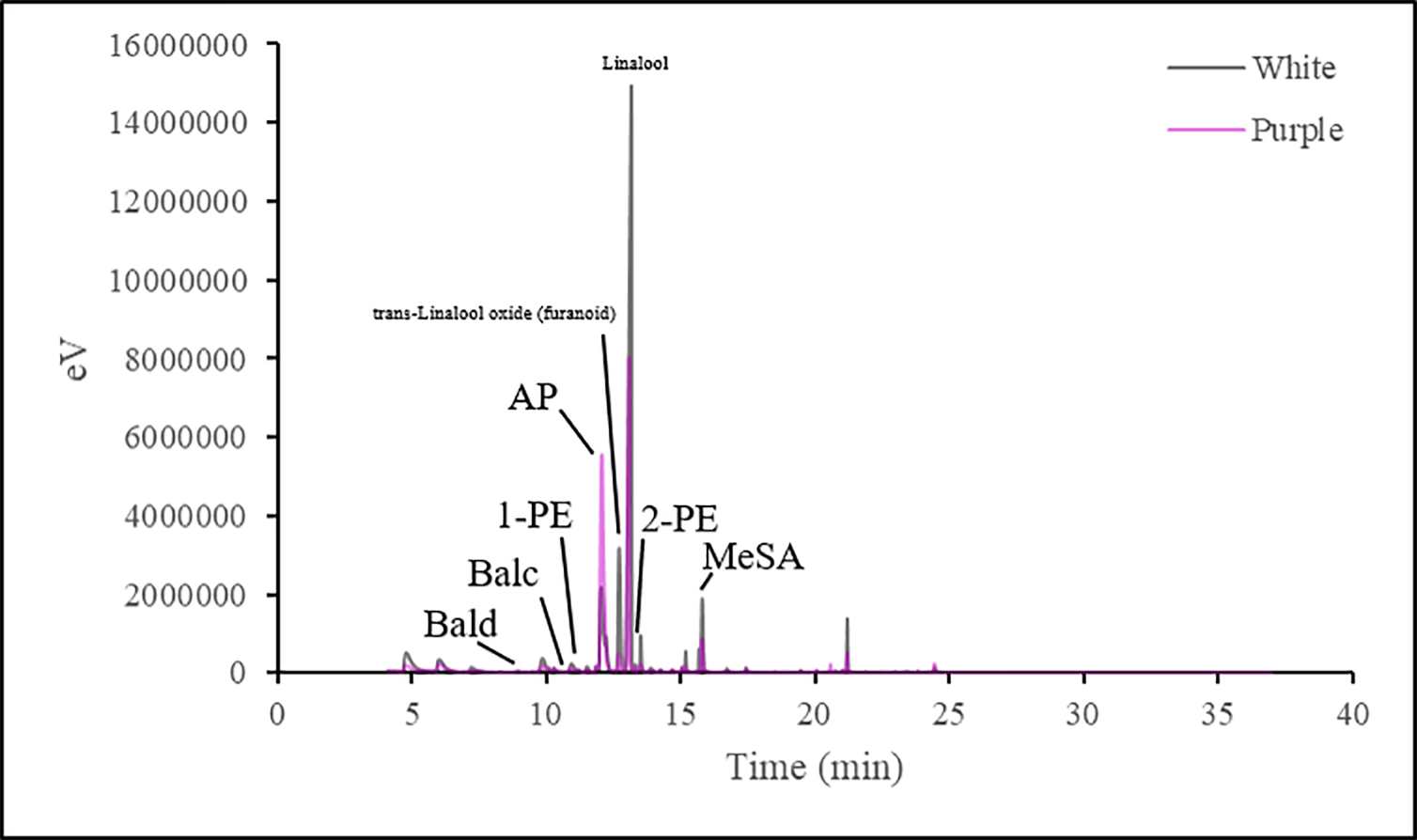

Supplement: Supplementary Figure 1 — Total ion chromatogram (TIC) of tea flower from gas chromatography-mass spectroscopy (GC–MS). [file Image_1.TIF]
